# Advancing antimicrobial strategies for managing oral biofilm infections

**DOI:** 10.1038/s41368-019-0062-1

**Published:** 2019-10-01

**Authors:** Yang Jiao, Franklin R. Tay, Li-na Niu, Ji-hua Chen

**Affiliations:** 1 Department of Stomatology, the 7th Medical Center of PLA General Hospital, Beijing, PR China; 2 Department of Endodontics, the Dental College of Georgia, Augusta University, Augusta, GA USA; 3 State Key Laboratory of Military Stomatology & National Clinical Research Center for Oral Diseases & Shaanxi Key Laboratory of Oral Diseases, Department of Prosthodontics, School of Stomatology, the Fourth Military Medical University, Xi’an, PR China

**Keywords:** Infection control in dentistry, Preventive dentistry, Infection control in dentistry, Preventive dentistry

## Abstract

Effective control of oral biofilm infectious diseases represents a major global challenge. Microorganisms in biofilms exhibit increased drug tolerance compared with planktonic cells. The present review covers innovative antimicrobial strategies for controlling oral biofilm-related infections published predominantly over the past 5 years. Antimicrobial dental materials based on antimicrobial agent release, contact-killing and multi-functional strategies have been designed and synthesized for the prevention of initial bacterial attachment and subsequent biofilm formation on the tooth and material surface. Among the therapeutic approaches for managing biofilms in clinical practice, antimicrobial photodynamic therapy has emerged as an alternative to antimicrobial regimes and mechanical removal of biofilms, and cold atmospheric plasma shows significant advantages over conventional antimicrobial approaches. Nevertheless, more preclinical studies and appropriately designed and well-structured multi-center clinical trials are critically needed to obtain reliable comparative data. The acquired information will be helpful in identifying the most effective antibacterial solutions and the most optimal circumstances to utilize these strategies.

## Introduction

Biofilm-related infections pose a major problem in the society from both an economical and health perspective.^1,2^ Biofilms are defined as “aggregates of micro-organisms in which the associated cells are frequently embedded in a self­-produced matrix of extracellular polymeric substances (EPS) that are adherent to each other and/or a surface.^1^” The EPS matrix not only provides microorganisms with a multilayered scaffold in which most cells experience cell­-to-­cell contact, either in flocs or in surface­attached biofilms, but also creates a microenvironment that is different from other sites in terms of key environmental inputs known to affect microbial behaviors, including pH, redox, and nutrient availability.^3^ Compared with planktonic microorganisms, the microorganisms in mature biofilm show increased tolerance to antimicrobial agents.^4^ It is estimated that biofilms contain multiple microbial species that weigh as high as 10^8^–10^11^ cells g^−1^ wet weight.^5^ The classical biofilm lifecycle may be described as a multi-stage process involving microbial attachment, biofilm maturation, and biofilm dispersal.^6^ Strategies that can disrupt any stage of biofilm formation are considered potentially valuable in controlling biofilm-related infections.

The warm, moist, and nutritious oral environment provides an ideal hatchery for microbial growth and proliferation. The complex dynamic interactions among microorganisms, host and diet result in microbial colonization and the subsequent formation of pathogenic biofilms. Biofilms formation on either tooth or dental material surfaces, known as oral biofilms, have been clearly recognized as a virulence factor in many oral infectious diseases, including dental caries, periodontitis and endodontic infections.^7,8^ Restorations, non-surgical or surgical periodontal therapies, root canal therapy, and dental implants are well-accepted therapeutic regimens, but secondary biofilm infections cannot be completely eliminated. Because of the increased drug tolerance, the complexity of the oral cavity and the rapid clearance of saliva, topical application of antimicrobial agents cannot be maintained at an effective concentration at the site of interest for a long enough period. The consequences of these infections depend on the location of biofilms and features of dental materials.^9^ Acid production by biofilms at the tooth-restoration margin causes secondary caries, which is a main reason for restoration failures. Pulp infections have also been clinically observed after dental restorations. Persisting biofilms inside the root canal system after root canal therapy may result in re-infections and persistent apical periodontitis. Biofilms on periodontal tissues and dental implants may cause periodontitis and peri-implantitis. The long-term clinical success of oral rehabilitation procedures depends on the capacity of dental materials to incorporate specific antimicrobial strategies for controlling and/or eradicating these infections. The present review encompasses a critical appraisal of recently published, innovative antimicrobial strategies for controlling oral biofilm-related infections.

## Antimicrobial dental materials

Antimicrobial materials to prevent bacterial adhesion and biofilm formation are of increasing importance because of the significant burden derived from surface-associated infections. Extensive efforts have been made to render dental materials with antimicrobial property. There are three major strategies: antimicrobial agent release, contact-killing, and multi-function^10^ (Table 1). Representative antimicrobial agents used for these strategies and their modes of action are listed in Table 2.

**Table 1:** Summary of strategies for antimicrobial dental materials

Name	Advantages	Drawbacks
Antimicrobial agent release (locally)	Strong and broad-spectrum antimicrobial activity, high local doses at site of interest, less systemic toxicity, and minimize the risk of antimicrobial resistance	Reservoir exhaustion issue, and short-acting
Contact-killing	Long-term antimicrobial activity, non-toxic and non-irritant properties	Surface biofouling, bacteriostatic effect (most)
Multi-functional strategy	Multiple functions (e.g., antibacterial, antifungal, antiviral, remineralizing, protein-repellent properties)	Selection of more combinations for synergistic antimicrobial and beneficial properties

**Table 2:** Representative antimicrobial agents and their mechanisms of action (modified from ref. ^22^)

Material type	Representative compounds	Mechanisms of action	Reference
Antibiotics	Aminoglycosides (e.g., gentamicin, tobramycin)	Bind to the bacterial 30S ribosomal subunit and inhibit protein synthesis	^12^
	Glycopeptides (e.g., vancomycin)	Bind to amino acids and disrupt cell wall peptidoglycan synthesis	
	Penicillins (e.g., ampicillin)	Inhibit related enzymes and disrupt cell wall peptidoglycan synthesis	
	Quinolones (e.g., ciproflaxin, norfloxacin)	Inhibit DNA replication and transcription, targeting DNA topoisomerases II and IV	
	Rifamycins (e.g., rifampin)	Bind to RNA polymerase and inhibit transcription	
	Tetracyclines (e.g., minocycline, tetracycline)	Inhibit protein synthesis	
Antimicrobial enzymes (AMEs)	Lysozyme	Catalyze glycosidic bond hydrolysis in bacterial cell wall peptidoglycans	^10^
	Acylase	Quorum-quenching	
Antimicrobial peptides (AMPs)	Natural AMPs (e.g., human β-defensin 1–3, magainin and nisin)	Transmembrane pore formation, intracellular targeting and metabolic inhibition mechanisms (inhibition of microbial functional proteins, DNA and RNA synthesis)	^48, 156^
	Synthetic AMPs (e.g., β-17, human neutrophil peptides 1 and 2, histatins 5 and 8)		
Cationic compounds	Chitosan	Interaction between positively charged chitosan molecules and negatively charged bacterial cell membranes leads to disruption of cell membrane	^157^
	Chlorhexidine	Bind to negatively charged bacterial walls and disrupt cell walls	
	Poly(ε-lysine)	Electrostatic adsorption onto bacterial cell membranes and stripping of the outer membrane, resulting in cell death	
	Quaternary ammonium compounds (QACs)	Disruption of bacterial enzymes and cell membranes by positively charged polymers	
Metal and metal oxides	Ag NPs	Induce oxidative stresses, deactivate bacterial enzymes by binding to thiol groups and affect the function and permeability of the cell membranes	^14, 158^
	Cu NPs	Contribute to ROS formation and induce lipid peroxidation in bacterial membranes	
	TiO_2_ NPs	Photocatalytically activate the production of ROS and interfere with phosphorylation, thereby causing oxidative cell death	
	ZnONPs	Generate ROS and binds to lipids and proteins, thus changing the osmotic balance and increasing membrane permeability	
Other non-cationic compounds	Nitric oxide (NO) donors	Induce cellular nitrosative and oxidative stresses and act as a bacterial signaling disruptor	^159, 160^
	Triclosan	Deactivate bacterial fatty acid biosynthesis through inhibition of the enoylacyl carrier protein reductase enzyme	^161, 162^
Natural products	Tea (e.g., tea catechins)	Irreversible damage to the microbial cytoplasmic membrane, inhibit the activity of salivary amylase, leading to reduced cariogenicity of starch-containing foods	^19^
	Propolis (e.g., trans–trans farnesol)	The lipophilic moiety interaction with bacterial membrane	^163^
	Cranberry (e.g., proanthocyanins, flavonol)	Inhibition of biofilm formation to prevent bacterial coaggregation, reduction of bacterial hydrophobicity, and alternation of cell surface molecules	^164^
Amino acids	Arginine	Counter the acid stress imposed by acidogenic bacteria and maintain a healthy oral biofilm	^3^
Antioxidants	N-acetylcysteine (NAC)	Inhibit bacterial cysteine, react with bacterial cell proteins, reduce bacterial extracellular polymeric substances, and disturb intracellular redox equilibrium	^152^

*NPs* nanoparticles, *ROS* reactive oxygen species

### Antimicrobial agent release

The idea of producing release-based antimicrobial materials dates back to 1950s, when Colton et al. incorporated antibiotic drugs into dental cements and resins.^11^ This strategy has since attracted considerable attention. These materials exert their antimicrobial activity by releasing preloaded antimicrobial agents over time into the environment to kill bacteria.^12^ Compared with systemic drug delivery, local delivery of antibiotics from the material surface offers advantages including high local doses of antimicrobial agents at a specific site without exceeding systemic toxicity and thus minimizing the development of resistance.^12^ The first generation of release-based agents are mainly antibiotics or silver compounds.^13^ Silver compounds are effective against biofilms even at a very low concentration to prevent bacterial growth. Their strong and broad-spectrum antimicrobial characteristics involve the induction of oxidative stresses, deactivation of bacterial enzymes by binding to thiol groups, and an increase in the permeability of bacteria cell membranes.^14^ Nevertheless, these release-based systems suffer from their inherently limited reservoirs of antimicrobial agents, and lack of long-term properties. Dental materials evolve with the introduction of nanotechnology either through the development of new materials or improvements in the properties of existing materials.^15,16^ The unique properties of nanoparticles (NPs), such as their small size, high surface area, and capability of releasing high levels of ions at low incorporated amounts, distinguish them from the same materials in micro-size or bulk size.^17^ Many nanomaterials, such as Ag, Cu, TiO_2_, ZnO, chitosan, and quaternary ammonium polyethylenimine (QPEI) NPs have been effective in controlling biofilms and incorporated into polymer matrices as filler particles.^14^

Apart from conventional biocidal antimicrobial agents, researchers devised new methods of combining dental materials with bacterial signaling pathway (e.g. quorum-sensing) inhibitors. The latter include NO donors,^18^ natural products such as tea,^19^ and antioxidants such as N-acetylcysteine,^20^ all of which have been shown to limit bacteria adhesion and biofilm formation. Bacterial attachment and subsequent formation of biofilm on surfaces result in changes in the local microenvironment, such as reduction in pH levels and release of bacterial products (e.g. specific enzymes) or virulence factors.^3^ The recently developed “smart” drug-delivery systems encompass “turn on” biocidal activity in response to a bacteria-containing microenvironment. This is achieved either by exposure of the surface-bound biocidal groups or release of preloaded antibiotics.^21^ For example, pH-activated polymeric NPs loaded with farnesol are capable of releasing the active drug at cariogenic acidic pH (4.5–5.5). These novel NPs effectively disrupted *S. mutans* biofilm and attenuated biofilm virulence in an in vivo dental caries model.^22^ Nevertheless, release-based antimicrobial dental materials still require extensive investigations before they may be recommended for clinical applications. Innovative strategies for controlling release kinetics within the therapeutic window, balance between the ability to kill bacteria and cytotoxicity to eukaryotes, and long-term effectiveness in case of secondary infections are desirable and ultimately govern the success of these materials.

### Contact-killing

**Fig. 1:**
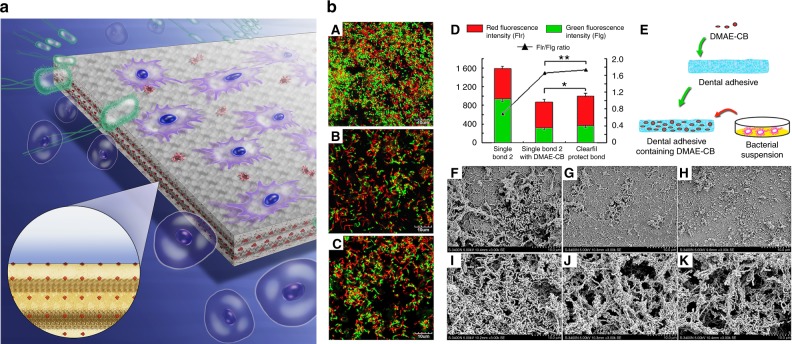
The application of antimicrobial dental materials. **a** Schematic illustration of antimicrobial dental materials. Proper incorporation of antimicrobial agents endows dental materials with antimicrobial function (dead microorganisms in red) and good biocompatibility (well-stretched viable cells in purple). **b** Antibacterial activity of polymerized dental adhesives containing MDPB or DMAE-CB. Representative confocal laser scanning microscopy images of *S. mutans* adhered on the control adhesive **A**, adhesive containing MDPB **B** and adhesive containing DMAE-CB **C** after 24-h incubation. Bacteria with integral membranes were stained with green fluorescence and those with compromised membranes were stained with red fluorescence. **D** Fluorescence intensity values of the two channels for adhesives derived from the three groups. **E** Schematic representation of polymerized adhesive containing DMAE-CB. Representative scanning electron microscopy images of *S. mutans* biofilms on the control adhesive **F**, adhesive containing MDPB **G**, and adhesive containing DMAE-CB **H** after 4-h incubation. Representative scanning electron microscopy images of *S. mutans* biofilms on the control adhesive **I**, adhesive containing MDPB **J**, and adhesive containing DMAE-CB **K** after 24-h incubation.^22^ Copyright 2017. Reproduced with permission from Elsevier Ltd

To circumvent the reservoir exhaustion issue of release-based antimicrobial materials, contact-killing strategy utilizes antimicrobial agents that are covalently anchored to the polymer backbone (Fig. 1).^22^ In this strategy, the antimicrobial agents used range from synthetic chemicals, such as quaternary ammonium compounds (QACs) and polycations to natural biomolecules such as antimicrobial peptides (AMPs).^23^

The use of QACs as antiseptics and disinfectants has a history of nearly one century. To date, the worldwide consumption of QACs is reported to exceed 0.7 million tons.^24^ Structurally, QACs are composed of nitrogen (N^+^)-containing compounds in which the N atom is attached to four different groups by covalent bonds. The representative formula is N^+^R_1_R_2_R_3_R_4_X^−^, where R can be a hydrogen atom, a plain alkyl group or an alkyl group substituted with other functionalities, and X represents an anion, which is most often a halide anion, although in some preparations, a hydroxyl anion may be involved. QACs are cationic surfactants and antimicrobials with broad-spectrum and strong contact-killing activity toward both Gram-positive and Gram-negative bacteria, fungi, malaria, amoebas, and viruses.^25^ The antimicrobial effect of QACs has been perceived as a function of the equilibrium among multiple factors including molecular weight, length of the N-alkyl chain and counter anions.^23^ Although not fully elucidated, the antimicrobial mechanism of QACs is generally believed to be attributed to disruption of the bacterial cell membrane structures.^26^ There are three methods to synthesize polymeric materials with pendant QACs. The first involves quaternization of polymers containing either tertiary ammonium groups or alkyl halides. The shortcomings of such an approach are the unpredictable impact of neighboring groups and limited degree of quaternization due to steric hindrance. Preparation of polymerizable quaternary ammonium monomers (QAMs), which are subsequently polymerized or copolymerized into a polymer network, has been developed for fabrication of QA-based dental materials. Another method is to incorporate QAC as a filler particle, such as the well-known QPEI NPs.^27,28^ The first synthetic QAM to be incorporated in antibacterial dental materials is 12-methacryloyloxy dodecyl pyridinium bromide (MDPB), which was commercialized as an antibacterial adhesive system (Clearfil Protect Bond^TM^, Kuraray Noritake Dental Inc., Tokyo, Japan).^29,30^ Unpolymerized MDPB exhibits strong antibacterial effects on various oral bacteria and biofilms. The minimum inhibitory concentration (MIC) and minimum bactericidal concentration (MBC) of MDPB against the seven cariogenic oral streptococci range from 31.3 μg·mL^−1^ to 62.5 μg·mL^−1^.^31^ Compared with MDPB (15.6/62.5 μg·mL^−1^), unpolymerized methacryloxyl ethylcetyldimethylammonium chloride (DMAE-CB) exhibits lower MIC/MBC (3.91/7.81 μg·mL^−1^) values against *S. mutans*. DMAE-CB also demonstrates potent killing effects at relatively lower concentrations than MDPB against both planktonic and adhering bacteria; this result is indicative that DMAE-CB has stronger antibacterial activity.^32,33^ After curing, QAM-based dental materials such as resin composites, dental adhesives, glass ionomer cements (GICs), and resin-modified GICs, pit-and-fissure sealants, pulp capping materials, root canal sealers and acrylic resins exhibit “contact inhibition” effects on bacteria that contact their surfaces.^34^ Other polymerizable QAMs have been synthesized and their antibacterial activities have been investigated in previously published reports, using various bacteria strains.^35,36^ The first generation of QAMs are mono-methacrylates with only one methacrylate group, which unavoidably limits their incorporation into resin polymer network structures (5 wt% MDPB and 3 wt% DMAE-CB in adhesives). Incorporating high concentrations of mono-methacrylates beyond the polymerizable capability of resin polymer network may inevitably affect their structures and mechanical properties. By harnessing the groundbreaking advancements in organic chemistry and materials science, QAMs with dimethacrylate groups have been synthesized to enhance their polymerization within a resin network. For example, 2-methacryloxylethyl dodecyl methyl ammonium bromide (MAE-DB) and 2-methacryloxylethyl hexadecyl methyl ammonium bromide (MAE-HB) have been synthesized. These QAMs with dimethacrylate groups exhibit strong bactericidal activity, as evidence by their considerably lower MBC values against eight species of oral bacteria, ranging from 12.2 μg·mL^−1^ to 24.4 μg·mL^−1^ for MAE-DB, and 6.2 μg·mL^−1^ to 48.8 μg·mL^−1^ for MAE-HB.^37,38^ Likewise, dental materials containing bis(2-methacryloyloxy-ethyl) dimethyl-ammonium bromide, which has a low viscosity and can be easily mixed with common dimethacrylates, exhibited strong anti-bacterial/anti-biofilm ability and the antibacterial effects may be retained for 6 months. The improved antimicrobial activities are attributed to the incorporation of more antibacterial resin monomers in the resin composites (18 wt%) and adhesives (10 wt%).^39,40^ In an investigation of the structure–property relationship of QAMs, those with a chain length of 12 or 16 were found to possess higher antibacterial potency.^41^ Moreover, changing the position of the functional groups in QAMs alters their anti-caries effects after their incorporation into dental resin.^42^ The combined use of augmented pressure adhesive displacement (0.3 MPa vs. conventional 0.1 MPa air-spray) and an experimental antibacterial adhesive containing 10 wt% MAE-DB significantly improved the caries resistance property of bonded dentin, reduced the progression of artificial carious lesions, and did not cause irreversible pulpal damage to vital teeth.^43,44^ Apart from in vitro models, experimental data from in vivo animal experiments also demonstrated that QAM-based dental materials exhibited potent antimicrobial efficiency and excellent biocompatibility.^45,46^

AMPs are a wide-ranging class of host-derived endogenous biomolecules that play important roles in innate immunity.^47^ Due to their broad-spectrum antimicrobial activities and low risks of developing antimicrobial resistance, they represent alternatives to traditional synthetic antimicrobial agents for developing antimicrobial materials.^48^ The most-widely accepted mechanism of AMPs for killing microorganisms is membrane permeabilization and disruption, as well as intracellular targeting. The latter includes inhibition of microbial functional proteins, DNA and RNA synthesis, interactions with certain intracellular targets following uptake through direct penetration and endocystosis.^49,50^ Apart from naturally occurring AMPs, researchers also identified AMPs from databases and developed synthetic AMPs with unique properties.^51^ These peptide mimics share the bactericidal mechanism of natural AMPs, but overcome their limitations, such as instability to enzymatic degradation, susceptibility to proteolysis and hydrolysis, hemolytic activity and cytotoxicity, as well as their ineffectiveness in eradicating multidrug-resistant bacteria.^52^ Novel technologies have been developed for manufacturing antimicrobial materials immobilized with AMPs on their surface.^53^ Table 3 represents some contact-killing Ti implants immobilized with AMPs. For example, calcium phosphate-AMP (Tet213, KRWWKWWRRC)-coated implants (by electrolytic deposition technique) possessed antimicrobial activity against both Gram-positive *Staphylococcus aureus* and Gram-negative *Pseudomonas aeruginosa* within 30 min in vitro.^54^ Amphipathic AMPs GL13K and D-GL13K were used to directly coat dentin before the restorative procedures. The modified dentin-composites resisted in vitro hydrolytic, mechanical, thermal, acidic, and enzymatic modes of degradation. Both the AMPs and the modified dentin-composites are active against multispecies biofilms derived from caries-active individuals.^55^

**Table 3:** Representative contact-killing Ti implants immobilized with AMPs

AMPs	Coating methods	Microorganisms or models tested	Reference
Bacitracin	Surface tethering	*Staphylococcus aureus* in vitro and in a rat femur implant infection model	^165^
GL13K	Surface tethering	*Streptococcus gordonii* and *Porphyromonas gingivalis* in vitro	^166^
GZ3.27	Surface tethering	*Pseudomonas aeruginosa* and *Escherichia coli* in vitro	^167^
hLF1-11	Polymer brush	*Streptococcus sanguinis* and *Lactobacillus salivarius* in vitro	^168^
Melimine	Surface tethering	*Pseudomonas aeruginosa* in vitro, and *Staphylococcus aureus* in mouse and rat subcutaneous implant infection	^169^
SESB2V	Surface tethering	*Staphylococcus aureus* and *Pseudomonas aeruginosa* in a rabbit keratitis model	^170^
Tet20	Polymer brush	*Pseudomonas aeruginosa* and *Staphylococcus aureus* in vitro, and *Staphylococcus aureus* in rat subcutaneous implant infection model	^171^
Tet213	Electrolytic deposition	*Pseudomonas aureus* and *Pseudomonas aeruginosa* in vitro	^54^
Tet213	Polymer brush	*Pseudomonas aeruginosa* in vitro	^172^

*AMP* antimicrobial peptide, Ti titanium

There are still major issues related to contact-killing antimicrobial materials. The antimicrobial activity of these materials is critically dependent on the chemical tethering procedure and the orientation of the covalently attached antimicrobial agents. Therefore, the antimicrobial activity of the resulting coating may not be as strong as those in free form. Most of these materials show antimicrobial activity via direct contact with bacteria on the immobilized antibacterial surface, without effects on planktonic bacteria.^56^ These materials only exhibited bacteriostatic instead of bactericidal effects against the contacting bacteria. This phenomenon may be attributed to inadequacy of surface-active functional groups derived from the antimicrobial agents in killing bacterial cells on contact. Another realistic issue with contact-killing antimicrobial materials is “surface biofouling”; Dead bacterial cells or saliva-derived protein films can also be absorbed onto the surface of the antimicrobial materials, blocking functional antibacterial groups on the material surfaces, hence inactivating and reducing the antimicrobial activity of those materials.^57^ However, most studies of this field do not investigate the half-life for antimicrobial efficacy. It is noted that cationic polymers, QACs and metal NPs are potentially toxic to eukaryotic cells and tissues.^58,59^

### Multi-functional strategy

Although strategies involving the release of antimicrobial agents and contact-killing have been developed, neither of these strategies has been entirely successful due to their inherent drawbacks. The biological environment within human body is complex, and antimicrobial materials with additional specific properties (e.g. remineralization or protein-repellent properties) are necessary to achieve better performance in their environments. Recently, several multifunctional antimicrobial materials have been developed to achieve augmented antimicrobial or multi-functional applications.^60,61^ Herein, several selected examples will be presented.

Silver has antibacterial, antifungal, and antiviral capabilities. Compared with free Ag, Ag NPs have a high surface area-to-mass ratio, and it is easier to control their releasing kinetics and to maintain long-term antibacterial effects.^62^ Experimental dental adhesives and composites containing AgNPs at a very low concentration are effective against plaque biofilms.^63,64^ To further enhance the antibacterial effects, the combined use of QAMs and AgNPs have recently been adopted for the preparation of antimicrobial dental materials. The modified materials effectively kill bacteria both on the surface of the materials and away from the surface.^65^ In addition, AgNPs are small enough (with a mean diameter of 40 nm) to penetrate patent dentinal tubules to kill residual intratubular bacteria.^66^ For the application of AgNPs in dentistry, some fundamental aspects need be taken into consideration: (1) the size of AgNPs should be small enough (smaller than 50 nm) to effectively penetrate the biofilm;^67^ (2) The incorporated amounts of AgNPs exhibit an inverse relationship with the original properties of carrier materials. Up to 0.1 wt% AgNPs does not significantly reduce microtensile bond strength and dentin shear bond strength of experimental adhesives,^64^ or the flexural strength of resin composites.^68^ However, 0.15 wt% or higher amount of AgNPs in experimental adhesives may cause a decrease in dentin bond strength.^65^ Moreover, as the incorporated AgNPs increased, the color of the resins become darker due to their plasmon effect,^69^ and 0.175 wt% addition gives the composite a brownish color;^70^ And (3) a growing concern exists regarding the toxicity of AgNPs, as well as other NPs used in dentistry to human beings.^71^ Similar to QACs, silver has also been successfully paired with other antimicrobial agents, such as antibiotics, metals, and NO donors.^72–74^

The use of NPs of amorphous calcium phosphate (NACPs) is helping in restoring the de-/re-mineralization balance, and imitates the biomineralization process of dentin and remineralizes tooth decay.^75^ Compared with micro-sized particles, NACPs have better ion-release profiles, and are more easily transformed into crystalline phases, such as octacalcium phosphate and apatite as a result of microcrystalline growth.^76,77^ Recent studies have incorporated both QAMs and NACPs into various kinds of dental materials. In in vitro studies, a NACP-QAM-modified nanocomposite inhibited biofilm and increased Ca and PO_4_ ion release at a low cariogenic pH. At this pH value, Ca and PO_4_ ions would be most needed for inhibiting caries, neutralizing lactic acid produced by the bacteria and preventing caries formation.^78^ An orthodontic adhesive containing QAMs and NACPs exhibited antibacterial and re-mineralization capabilities without adversely affecting bond strength, compared with the control. This antibacterial orthodontic adhesive represents a promising candidate in combating enamel white spot lesions and even dental caries.^79^ Strong antibacterial capabilities, as well as the original mechanical properties, were maintained after a 180-day water-aging process.^78^ Using a rat tooth cavity model, it was found that the QAM and NACP-modified nanocomposites and adhesives facilitated healing of the dentin–pulp complex with milder pulpal inflammation and more tertiary dentin formation compared with the control groups.^80^

Compared with antimicrobial materials that have a permanent and single killing mechanism, multi-functional materials exhibit several advanced properties to activate bactericidal activity in response to the microenvironment of bacterial infection.^61^ It is impossible to directly compare the effectiveness of multi-functional strategies across different studies. Therefore, studies on comparative effectiveness with a broader range of antimicrobial agents will be necessary to select more combinations with synergistic and enhanced antimicrobial properties for developing novel antimicrobial dental materials.

## Antimicrobial photodynamic therapy (APDT)

APDT has a history of more than 100 years, when Raab used acridine hydrochloride and visible light to inactivate *Paramecia caudatum*.^81^ Recently, APDT has been used increasingly in dentistry for the treatment of dental caries, periodontal and endodontic diseases, due to its potential disinfecting effect on various oral microbial pathogens and biofilms.^82^ The working principle of APDT involves the interaction between a photosensitizer and low-energy laser light in the presence of oxygen, to generate reactive oxygen species (ROS). The bactericidal effect of APDT is attributed to oxidative damage of bacterial DNA and cell membrane system.^83,84^ Depending on the type of agents, photosensitizers may be delivered in various ways including intravenous injection, oral ingestion or topical application.^85^ It is unlikely that microorganisms would develop antimicrobial resistance to APDT due to the primitive molecular nature of ROS. Moreover, the benefits of APDT include instant suppression of causative oral bacteria, absence of systemic disturbance and undesirable effects on healthy oral cells and tissues.

Dental caries is a breakdown of teeth due to acids generated by oral microbial biofilms.^22^ The APDT technique may be used to prevent dental caries by facilitating the dispersion of formed biofilms and eliminating pathogens within carious lesions.^86^ The use of APDT offers several proposed advantages, including rapid cariogenic bacterial killing, non-invasive nature to non-carious lesions and minimum antibiotic resistance. Accumulating experimental evidences have demonstrated the susceptibility of cariogenic bacteria, either in planktonic form or as biofilms, to APDT.^87,88^ For instance, the photosensitizer toluidine blue O-induced APDT has been shown to be effective against *S. mutans* in decayed teeth.^89–92^ Recently, the combined use of APDT and other treatment, such as a dental plaque-disclosing agent erythrosine^93^ or casein phosphopeptide-amorphous calcium phosphate achieved cariogenic bacteria killing in vitro and root surface caries arresting in vivo.^94^ Nevertheless, negative results have also been reported. The use of an APDT procedure with methylene blue as the photosensitizer demonstrated limited efficacy on killing of cariogenic bacteria in an in vitro multi-species biofilm model.^95^ Accordingly, more studies, especially randomized clinical trials (RCTs), are required to validate the antimicrobial effectiveness of APDT and to optimize its treatment parameters.

Periodontal diseases are the most common infectious diseases in humans that result in inflammatory destruction of the periodontium (i.e. gingiva, periodontal ligament, cementum, and alveolar bone). The primary etiological factor of periodontal diseases is the accumulation of microbial biofilms on tooth and root surfaces, which, in turn, induces an imbalance between the pathogenic bacteria and the host immunological potential.^96^ Scaling and root planning (SRP) is regarded as the gold standard treatment modality for non-surgical periodontal treatment.^97^ Nevertheless, SRP is associated with several limitations, including the inability to completely debride root surfaces and incomplete elimination of pathogenic bacteria in deep periodontal pockets and/or inaccessible furcation defects.^98^ Antimicrobial agents have also been used, but their use is not free of risks for drug resistance.^99^ Moreover, because of the rapid clearance of saliva, locally applied antimicrobial agents are not maintained at the therapeutic concentrations for a long enough duration.^100^ To surmount these limitations, certain adjunctive therapies, such as APDT have been proposed for periodontal diseases.^83^ Several studies have shown that periodontal pathogens are susceptible to APDT in planktonic cultures,^91^ plaque scrapings^92^ and biofilms,^101^ using different kinds of photosensitizers. In addition, the progression of periodontal diseases and destruction of periodontal tissues may be reduced significantly by the utilization of APDT.^102^ The APDT technique has been reported to be superior to the systemic antibiotic metronidazole in experimental animal models of periodontitis.^103^ The technique showed comparable effects as SRP in terms of clinical parameters,^104^ as well as the titer of tumor necrosis factor-alpha and receptor activator of nuclear factor-kappaB ligand (RANKL) in the gingival crevicular fluid of patients suffering from aggressive periodontitis.^105^ A meta-analysis of four RCTs indicated that a combined therapy of SRP and APDT provided additional clinical improvement in the maintenance of residual pockets during supportive periodontal therapy.^106^ Despite encouraging results, the longest follow-up period for the four RCTs is only 12 months. Indeed, more well-performed and long-term RCTs are still needed. In other studies using APDT as an adjunct to SRP, no beneficial effects over SRP alone were observed, which might be attributed to the inadequate exposure time for evaluating the clinical benefits.^107^ In addition, oral bacteria in biofilms was found to be less affected by methylene blue-induced APDT than planktonic bacteria.^108^ The reduced susceptibility might be due to inactivation of methylene blue, and protected phenotypes expressed by biofilm species on the agar surface.^109^ These controversies among different studies must be addressed with more innovative study design in future investigations.

APDT has also been employed recently for targeting microorganisms in root canal systems in vitro^110^ and in vivo.^111^ During APDT treatment in the root canal system, it is possible that the photosensitizers may pass into the periapical tissues through the root apex. This iatrogenic phenomenon may adversely affect the health status of periapical host cells after a photosensitizer is activated by light. Accordingly, it is important to determine the therapeutic window to ensure that bacteria are eliminated while host cells are left intact. To date, several in vitro studies have investigated the safety of APDT.^112^ The commonly used photosensitizer methylene blue ranging from 10 μmol·L^−1^ to 100 μmol·L^−1^ caused up to 36% and 100% killing for fibroblasts and *E. faecalis*, respectively, after exposure to red light.^113^ More recently, APDT has been used for root canal disinfection in a clinical setting as an adjunct to standard endodontic treatment.^114^

Other dental applications of APDT include treatment for peri-implantitis^115^ and oral lichen planus,^116^ as well as the disinfection of acrylic denture surfaces.^117^ Despite promising results, several factors should be considered to obtain good therapeutic outcomes. These factors include the type of photosensitizer, adequate tissue penetration especially for deep injuries, and the combination of other treatment modalities with APDT. Recently, great attention has been paid to biodegradable polymeric materials and NPs carrying photosensitizer agents. These materials possess several advantages including: (1) increased binding between photosensitizers and bacteria to improve bactericidal efficiency, (2) reduced aggregation of photosensitizers, and (3) improved biocompatibility and biodegradability.^118^ The use of APDT effectively eliminated pathogenic microorganisms with few reported side effects.^84^ The major reported side-effect is the existence of a period of residual skin photosensitivity owing to photosensitizer accumulation, which may last for several days to weeks depending on the administered photosensitizer. Hence, patients should be instructed to avoid skin and eye exposure to bright light or sunlight until the photosensitizer is completely eliminated.^119^ Moreover, APDT has not been approved by the United States Food and Drug Administration for dental applications. For clinical trials, approval by the local institutional review board should be obtained prior to treatment. Universally applicable guidelines with standardized parameters should be produced in this field to direct clinical applications.

## Cold atmospheric plasma (CAP)

**Fig. 2:**
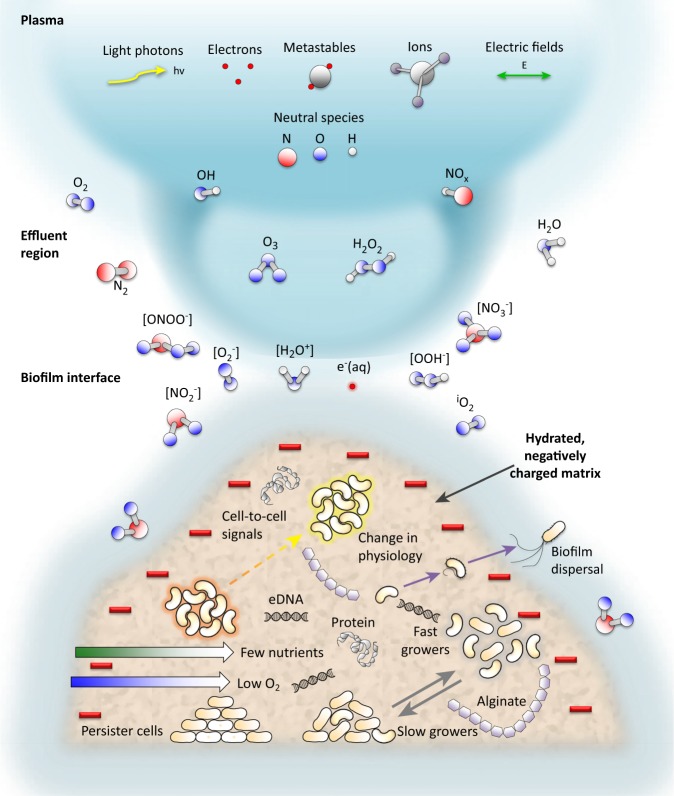
Antimicrobial mechanism for cold atmospheric plasma. The plasma-derived RONS that diffuse into the biofilm and cause oxidative damages to their cell membrane, DNA, and proteinaceous enzymes, resulting in disruption of the cell membrane and cell death. eDNA extracellular DNA, RONS reactive oxygen and nitrogen species.^120^ Copyright 2018. Reproduced with permission from Elsevier Ltd

CAP represents a promising non-antibiotic option for the eradication and control of biofilm infections.^120^ The CAP technique uses a highly reactive mix of ions and electrons, radical species, molecules in the ground or excited state and quanta of electromagnetic radiation (UV photons and visible light). Compared with conventional plasma technology, CAP is operated under atmospheric conditions, and is thus feasible for in vivo applications without damaging the surrounding tissues.^121^ Over the last few years, accumulating experimental evidences have demonstrated the efficacy of CAP in eliminating broad-spectrum microorganisms, including Gram-negative *P. aeruginosa* and *E. coli*, Gram-positive *S. aureus, B. subtilis*, and *M. luteus*, multi-drug-resistant species, such as methicillin-resistant *S. aureus*, fungi *T.* spp. and *C. albicans*, as well as for controlling bacterial biofilms.^122^ For example, CAP effectively reduce 94% of the regular bacterial skin flora^123^ and significantly reduced bacterial load in chronic wounds.^124^ Based on bacteriophage models, recent studies demonstrated that CAP is capable of reducing and inactivating human pathogenic viruses.^125^ Currently, the most widely accepted mechanisms of action for CAP are the occurrence of reactive oxygen and nitrogen species (RONS) as represented in Fig. 2.^126^ Most bacteria are sensitive to RONS which cause oxidative damages to their cell membrane, DNA, and proteinaceous enzymes.^127^ The role of charged particles, electrons, and ions in inactivation of bacteria has also been described. Charge accumulation on the bacterial membrane causes mechanical disruption of bacterial cell membrane.^128^ Recently, the CAP technology has been used in various dental applications including root canal treatment and dental implant surface modification.^129^

Microbial infection has long been recognized as the primary etiologic factor in the development of pulpitis and apical periodontitis.^130^ Root canal infection is normally treated with endodontic instruments, irrigation, ultrasonics, and the application of sodium hypochlorite and other antibacterial compounds. The purpose of root canal treatment is to completely eradicate all the microorganisms within the root canal system and to prevent re-infection.^131^ However, these traditional treatment methods cannot completely eliminate bacteria and bacterial biofilms, with remaining cultures ranging from 40% to 60%.^132^ Development of adjunctive strategies to enhance the effectiveness of traditional antimicrobial intracanal disinfection is advantageous for successful root canal therapy. A CAP microjet device with argon/O_2_ as the working gas was used to disinfect root canals in single-rooted extracted human teeth.^133^ It was found the inactivation rate of planktonic *E. faecalis* gradually increased with the treatment time and reached 98.8% after an 8-min CAP treatment. Root canals treated with CAP for 40 min showed no detectable re-infection. Indeed, *E. faecalis* is the most important pathogenic bacteria in non-healing apical periodontitis, as well as re-infection cases after root canal therapy.^134^ Compared with planktonic bacteria, mature intracanal biofilms are more resistant to commonly used intracanal medicaments, such as calcium hydroxide, which can damage the bacterial DNA due to its alkaline pH.^135^ Moreover, *E. faecalis* is alkali-resistant and cannot be killed by calcium hydroxide in the infected root canal.^136^ It has been demonstrated that CAP was effective not only for young biofilms,^137^ but also for mature *E. faecalis* biofilms.^138^ After a 10-min of CAP treatment, the regular structure of a 100-μm biofilm was destroyed and replaced with ruptured bacteria.^139^

By using various tooth-mimicking substrates,^140^ cavity models,^141^ dentin and enamel,^142^ CAP treatment demonstrated effective killing of various oral cariogenic bacteria, which are responsible for secondary caries formation and disruption of the durability of resin–dentin bonds. Oral single-species biofilm of *A. naeslundii*, *C. albicans*, *S. gordonii*, *S. mutans*, *S. oralis*, and *S. sanguinis* could be eradicated by twice daily application of CAP for 10–30 s each.^143^ Nevertheless, several studies discussed the limitations of CAP, regarding incomplete removal of biofilms.^144^ Biofilm thickness is a non-negligible factor; The polymeric matrix in biofilms may reduce CAP access, preventing its penetration into its deep layer and its bactericidal effect on bacteria at the bottom of the biofilms. In addition, tolerance of microbial pathogens and biofilms to CAP is variable between species, strains of the same species, and the composition of reactive species produced by different CAP devices. Nevertheless, CAP treatment has been shown to produce no harmful effects on the mucosa and periodontal tissues,^145,146^ thereby alleviating concerns on the potential adverse effect on health tissues when CAP is used for oral applications. Future studies should investigate the responses of dental pulp stem cells, which are crucial for reparative dentinogenesis after CAP treatment.

Microbial colonization and subsequent biofilm formation on the dental implants are a major causative factor of implant failure and peri-implantitis.^147^ Treatment with CAP significantly reduced the viability and quantity of in situ biofilms on Ti discs exposed to human oral cavities for 72 h, and its efficacy was found to be correlated with the treatment duration and plasma power.^148^ Importantly, no microstructural alterations of the Ti discs were observed after CAP treatment.^149^ The combined use of mechanical cleaning and subsequent CAP treatment improved osteoblast growth on biofilm-covered Ti discs. Cell growth on the Ti discs was comparable to the sterile control.^150,151^ Other investigations cautioned that complete elimination of biofilms could not be consistently achieved using CAP treatment only, and required supplementary application of other treatment modalities, such as air/water spray, air abrasion, or mechanical cleaning.^150^ Thus, CAP may provide adjunctive support for established decontamination techniques for treatment of peri-implantitis.

Although there is great potential for CAP to be adopted for clinical dental applications, several potential barriers to its clinical translation have been brought out. These include: (1) the effects of gas phase interactions with hydrated biological matrices, resulting in plasma-activated media; (2) a more precise definition of the plasma dose; (3) tissue-specific effects on the flux of species delivered; and (4) modulation of the host’s immune responses.^152^

## Summary and perspective

The past decade has witnessed the development of innovative strategies to control oral biofilm infections. Antimicrobial dental materials based on antimicrobial agent release, contact-killing, and multi-functional strategies have been developed for prevention of initial bacterial attachment and subsequent biofilm formation. Recent progress in controlled radical polymerization and click chemistry, which enables precise control over macromolecular structure, order, and functionality, has provided a powerful tool for the design and synthesis of bioactive surfaces and functional biomaterials, including antimicrobial dental materials.^153,154^ Novel experimental approaches for the management of microbial biofilms in clinical practice include the use of APDT as an alternative to antimicrobial regimes and mechanical removal of tenacious biofilms, and the use of CAP that shows potential advantages over conventional antimicrobial approaches.^120,155^

Despite the recent achievements that are summarized in the present review, it should be emphasized that many challenges remain. Most currently available data is obtained from in vitro experiments and in preclinical models in the short term. Researchers must provide quantitative evidences that these reported strategies bring real benefits under in vivo conditions and confirm their antibacterial longevity. The safety profile and development of antimicrobial resistance for these antimicrobial compounds and nanomaterials is a matter of overriding significance and needs further assessment. Appropriately designed and well-structured multi-center clinical trials are critically needed to obtain reliable comparative data to identify the most effective antibacterial solution and the most optimal parameters for utilizing these anti-biofilm strategies.
